# Intravenous and oral copper kinetics, biodistribution and dosimetry in healthy humans studied by [^64^Cu]copper PET/CT

**DOI:** 10.1186/s41181-020-00100-1

**Published:** 2020-06-18

**Authors:** Kristoffer Kjærgaard, Thomas Damgaard Sandahl, Kim Frisch, Karina Højrup Vase, Susanne Keiding, Hendrik Vilstrup, Peter Ott, Lars Christian Gormsen, Ole Lajord Munk

**Affiliations:** 1 Department of Hepatology and Gastroenterology, Aarhus University Hospital, Aarhus, Denmark; 2 Department of Nuclear Medicine & PET Centre, Aarhus University Hospital, Aarhus, Denmark

**Keywords:** ^64^Cu, Copper, Kinetics, Dosimetry, Biodistribution

## Abstract

**Purpose:**

Copper is essential for enzymatic processes throughout the body. [^64^Cu]copper (^64^Cu) positron emission tomography (PET) has been investigated as a diagnostic tool for certain malignancies, but has not yet been used to study copper homeostasis in humans. In this study, we determined the hepatic removal kinetics, biodistribution and radiation dosimetry of ^64^Cu in healthy humans by both intravenous and oral administration.

**Methods:**

Six healthy participants underwent PET/CT studies with intravenous or oral administration of ^64^Cu. A 90 min dynamic PET/CT scan of the liver was followed by three whole-body PET/CT scans at 1.5, 6, and 20 h after tracer administration. PET data were used for estimation of hepatic kinetics, biodistribution, effective doses, and absorbed doses for critical organs.

**Results:**

After intravenous administration, ^64^Cu uptake was highest in the liver, intestinal walls and pancreas; the gender-averaged effective dose was 62 ± 5 μSv/MBq (mean ± SD). After oral administration, ^64^Cu was almost exclusively taken up by the liver while leaving a significant amount of radiotracer in the gastrointestinal lumen, resulting in an effective dose of 113 ± 1 μSv/MBq. Excretion of ^64^Cu in urine and faeces after intravenous administration was negligible. Hepatic removal kinetics showed that the clearance of ^64^Cu from blood was 0.10 ± 0.02 mL blood/min/mL liver tissue, and the rate constant for excretion into bile or blood was 0.003 ± 0.002 min^− 1^.

**Conclusion:**

^64^Cu biodistribution and radiation dosimetry are influenced by the manner of tracer administration with high uptake by the liver, intestinal walls, and pancreas after intravenous administration, while after oral administration, ^64^Cu is rapidly absorbed from the gastrointestinal tract and deposited primarily in the liver. Administration of 50 MBq ^64^Cu yielded images of high quality for both administration forms with radiation doses of approximately 3.1 and 5.7 mSv, respectively, allowing for sequential studies in humans.

**Trial registration number:**

EudraCT no. 2016–001975-59. Registration date: 19/09/2016.

## Introduction

Copper is an essential mineral present in all tissues and is important for several enzymatic processes (Tapiero et al. 2003). It is absorbed in the upper intestine and taken up by the liver via the portal vein. From the liver, copper is either distributed to the systemic circulation bound to ceruloplasmin or, in the case of excess, excreted into the bile (Gupta and Lutsenko 2009). Disturbances in copper homeostasis are potentially fatal as seen in the rare genetic disorder of Wilson’s disease, where accumulation of toxic levels of copper in various organs leads to critical symptoms from the liver and central nervous system (Ala et al. 2007).

Human copper metabolism and kinetics are only partly understood despite recent advances in molecular imaging, notably positron emission tomography (PET). [^64^Cu]copper^1^ (^64^Cu) PET is characterized by high spatial resolution (positron range, 0.7 mm in water), and the radioactive half-life of ^64^Cu (*t*_1/2_ = 12.7 h) allows for in vivo assessment of copper biodistribution, even in compartments with slow copper turnover (Conti and Eriksson 2016). To date, only few ^64^Cu PET studies of biodistribution and radiation dosimetry after intravenous injection in humans have been published, but it remains unclear which organs are the most critical in terms of radiation exposure (Avila-Rodriguez et al. 2017; Capasso et al. 2015; Piccardo et al. 2018). In addition, no such studies have been published with oral administration of ^64^Cu, the natural entrance route of copper in humans.

^64^Cu PET/CT has been used in the context of cancer detection and characterization (Capasso et al. 2015; Piccardo et al. 2018; Panichelli et al. 2016; Wachsmann and Peng 2016), utilizing the overexpression of the human copper transporter 1 (CTR1) in malignant cells (Peng et al. 2006). To our knowledge, no studies have yet examined the potential of ^64^Cu PET/CT to assess temporal whole-body copper homeostasis in humans, in particular hepatic copper uptake, accumulation and excretion.

In this study, we determined the hepatic kinetics of ^64^Cu, characterized copper biodistribution and estimated the radiation dosimetry of ^64^Cu in healthy humans by sequential whole-body PET imaging spanning 1.5 to 20 h after intravenous as well as oral administration. The image based biodistribution estimates were supplemented by measurements of radioactive concentrations in blood, urine and faecal samples.

## Materials and methods

### Radiochemistry

Cyclotron-produced ^64^Cu (nuclear reaction: ^64^Ni(p,n)^64^Cu) was obtained from a commercial source (the Hevesy Laboratory, DTU Nutech, Risø, Roskilde, Denmark) and delivered to our centre as solid ^64^CuCl_2_ (radionuclidic purity ≥99%; specific activity ≥1.0 TBq/μmol) on the day of the study (one batch production of ^64^CuCl_2_ was used over 2 days for two or three participants). Before use, the received ^64^CuCl_2_ was dissolved in sterile 0.1 M HCl (1 mL), pH was adjusted to around 5 with sterile 0.5 M sodium acetate buffer (0.5 mL), and sterile saline (8.5 mL) was added. The acetate buffered ^64^Cu solution was finally passed through a sterilizing filter (0.22 μm) into a sterile product vial. Quality control of the ^64^Cu solution consisted of pH measurement (pH strips; specification: 4–6), radiochemical purity test (radio-TLC; specification: ≥95%), LAL-test (PTS Endosafe, Charles River Laboratories; specification: < 17.5 EU/ml), radionuclide identification (gamma spectrum; germanium detector; specification: 511 + 1346 keV), and sterile filter test (pressure-hold-test; specification: filter intact). The preparation and quality control of the ^64^Cu solution was approved by the Danish Medicines Agency.

### Study design and participants

Biodistribution and dosimetry for ^64^Cu after intravenous and oral administration were determined by dynamic liver and subsequent whole body PET/CT in six healthy human participants (age 22–61 years). Four participants received intravenous administration (IV1-IV4; two males, two females) and two received oral administration (O1-O2; one male, one female) of ^64^Cu (Table 1). In an additional four participants (IV5-IV8; 2 males, two females) blood, urine, and faecal samples were collected after intravenous ^64^Cu administration, but without PET imaging (Supplemental Table 1). Participants fasted for at least 6 h before administration of ^64^Cu, but were allowed to drink water. Study inclusion criteria were: Age above 18 years, and for females, negative pregnancy test and use of safe contraception. Criteria for exclusion were known hypersensitivity to ingredients in the formula, use of drugs that affect copper metabolism, history of clinical disease, current pregnancy, breastfeeding, or desire to become pregnant. No complications to the procedures were observed.

**Table 1:** Participant characteristics, gender-averaged absorbed dose estimates (μGy/MBq), and effective dose (μSv/MBq) for ^64^Cu by intravenous (IV) and oral administration

	IV				Oral	
*ID* (sex/age)	IV1 (M/61)^a^	IV2 (F/25)	IV3 (M/24)	IV4 (F/22)	O1 (F/39)	O2 (M/27)
BW/height (kg/cm)	76/178	75/175	94/186	68/160	54/168	77/181
Dose (MBq)	116.4	66.04	73.0	77.0	57.3	61.3
**Target organ**						
Liver	415.0	467.0	462.0	446.0	317.0	335.0
Gallbladder	87.8	108.0	126.0	68.4	144.0	119.0
Stomach	48.3	61.1	58.0	48.8	274.0	238.0
Small Intestine	188.0	238.0	191.0	168.0	369.0	395.0
RLI	225.0	88.7	181.0	213.0	925.0	600.0
LLI	250.0	87.1	120.0	121.0	30.4	375.0
Kidneys	137.0	128.0	133.0	132.0	66.0	72.6
Pancreas	116.0	122.0	110.0	173.0	51.8	51.5
Red Bone Marrow	36.2	34.0	35.5	32.5	27.0	24.4
**Effective Dose**	67.6	56.2	62.0	61.3	114.0	112.0

Data for critical target organs and effective doses for all individuals are displayed; for full list of organs, see Supplemental Table 3

*Abbreviations*: *BW* Body Weight, *RLI* Right Large Intestine, *LLI* Left Large Intestine

^a^Dynamic PET/CT scan and blood samples not obtained

### PET/CT acquisition

The participants were placed in supine position in a Siemens Biograph™ 64 TruePoint™ PET/CT camera within the 21.6 cm axial field-of-view. A low dose CT scan (50 effective mAs with CARE Dose4D, 120 kV, pitch of 0.8 mm, slice thickness 5.0 mm) was performed before each PET scan for definition of anatomic structures and attenuation correction of the PET images. The ^64^Cu solution was administered as an intravenous bolus injection (*n* = 4; median dose 73.5 MBq, range 66–116 MBq) or dissolved in water and swallowed (*n* = 2; median dose 65.5 MBq, range 57–74 MBq). All participants underwent a dynamic PET scan of 90 min (dynamic PET and blood sampling were not acquired for one participant, see Table 1) with field-of-view over the liver, recorded in list-mode; time frame structure was 12 × 5 s, 8 × 15 s, 7 × 60 s, and 16 × 300 s. This was followed by three consecutive whole-body PET/CT scans (top of skull to mid-thigh; 6 bed positions) performed at 1.5, 6, and 20 h after tracer administration (duration 6, 6, and 10 min per bed position). The PET images were reconstructed using 3-dimensional ordered-subset expectation maximization with 4 iterations and 21 subsets, 4-mm Gauss filter, and 168 × 168 matrix with voxel size 4 x 4 x 5 mm^3^.

### Image processing

The fused PET/CT images were analysed using the PMOD 3.7 software (PMOD Technologies Ltd., Zürich, Switzerland). For kinetic analysis, the time course of the activity concentration of ^64^Cu during the 90-min dynamic PET scan was measured in a volume-of-interest (VOI) placed in the right liver lobe. The VOIs were drawn to contain liver tissue while avoiding large intrahepatic blood vessels and bile ducts. For biodistribution and dosimetry calculations, all tissues were visually inspected on images of the 1.5 h, 6 h, and 20 h whole body scans by two investigators. Organs with accumulation of ^64^Cu above that of surrounding tissue were defined as source organs: liver, gallbladder contents, small intestine, left large intestine (descending and sigmoid colon), right large intestine (ascending and transverse colon), rectum, stomach contents, kidneys, pancreas (IV only), and red bone marrow. VOIs were manually drawn for each source organ to encompass all radioactivity of the respective organ. The red bone marrow activity was estimated based on VOIs in the lumbar vertebrae as described by McParland (McParland 2010).

### Blood, urine and faecal samples

In the intravenous study, arterial blood samples were collected from a radial artery during the initial dynamic PET scan at time points 12 × 5 s, 8 × 15 s, 7 × 60 s, and 16 × 300 s. In the oral study, venous blood samples were collected from a peripheral vein during the initial dynamic PET scan and before each of the consecutive whole-body scans (1.5 h, 6 h, and 20 h). In four additional participants with intravenous tracer administration (IV5–8), venous blood samples were obtained as for the oral study, and total urine and faeces were collected from 0-6 h and 6–20 h. Radioactivity concentrations of ^64^Cu were measured in whole blood, plasma, urine, and faeces using a well gamma counter (Packard 5003, Packard Instruments, USA). Time courses of the activity concentration in blood and plasma were generated for 90 min with two additional samples at 6 h and 20 h. Total output in percent of administered dose (%AD) for urine and faeces were calculated for time points 6 h and 20 h. All concentration measurements were cross-calibrated with the PET-camera and corrected for radioactive decay back to start of the tracer administration. Prior to the injection of radiotracer, a venous blood sample was drawn for measurement of baseline blood tests of liver and kidney function, haematological quantities, and copper metabolism.

### Modelling of hepatic kinetics

Kinetic parameters were estimated by fitting kinetic models to the dynamic liver PET data using the time course of arterial plasma ^64^Cu as input function. To account for the hepatic dual blood supply from the hepatic artery (25%) and portal vein (75%), we used reversible linearised models that allow robust and unbiased estimates using only the arterial input function (Munk et al. 2001). Two kinetic models were used: 1) The Gjedde-Patlak linearisation yielding the steady-state clearance from blood to liver tissue (*K*; mL blood/min/mL liver tissue) including a small reversible loss rate constant (*k*_loss_; min^− 1^), representing the loss of tracer from the hepatocytes into bile or blood (Patlak and Blasberg 1985); 2) the Logan linearisation that estimates the total distribution volume (*V*_d_; mL blood/mL liver tissue) of ^64^Cu in the liver (Logan et al. 1990). Both kinetic models were applied to data 30 to 90 min after tracer administration to ensure quasi-steady-state. The kinetic model parameters were estimated using software developed in-house (Supplemental Figure 1).

### Biodistribution and dosimetry

For each source organ, the time course of the non-decay-corrected total radioactivity was normalised to the administered activity and recalculated to time courses of percentage injected activity. Time-integrated activity coefficients (TIACs) were computed using the trapezoidal integration method to calculate the area under the curves, assuming only physical decay after the last scan without further biological clearance. The remainder TIAC was calculated by subtracting the individual source organ TIACs from the total body TIAC (without voiding), which for ^64^Cu is 18.3 h. TIACs for source organs and remainder were used in OLINDA/EXM 2.0 (HERMES Medical Solution AB, Sweden) (Stabin and Siegel 2018) to compute organ absorbed doses (μGy/MBq) and the effective dose (μSv/MBq) using anthropomorphic human body phantoms with organ masses based on ICRP89 (Basic anatomical and physiological data for use in radiological protection: reference values. A report of age- and gender-related differences in the anatomical and physiological characteristics of reference individuals. ICRP Publication 89 2002) and ICRP103 tissue weighting factors (The 2007 Recommendations of the International Commission on Radiological Protection 2007). Organ doses and effective dose results are given for the reference gender-averaged adult according to ICRP103.

## Results

### Biodistribution

**Fig. 1:**
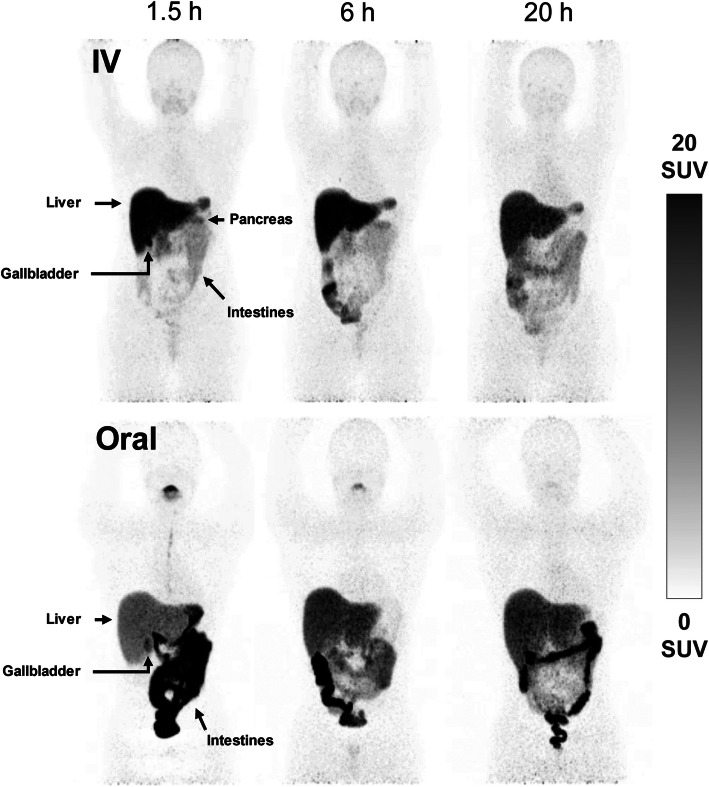
Whole-body PET images (maximum intensity projection) showing the biodistribution of ^64^Cu after intravenous (upper panels) and oral (lower panels) administration in two healthy individuals (IV4 and O2). PET imaging was performed 1.5, 6, and 20 h after administration. Arrows identify most visible source organs

**Fig. 2:**
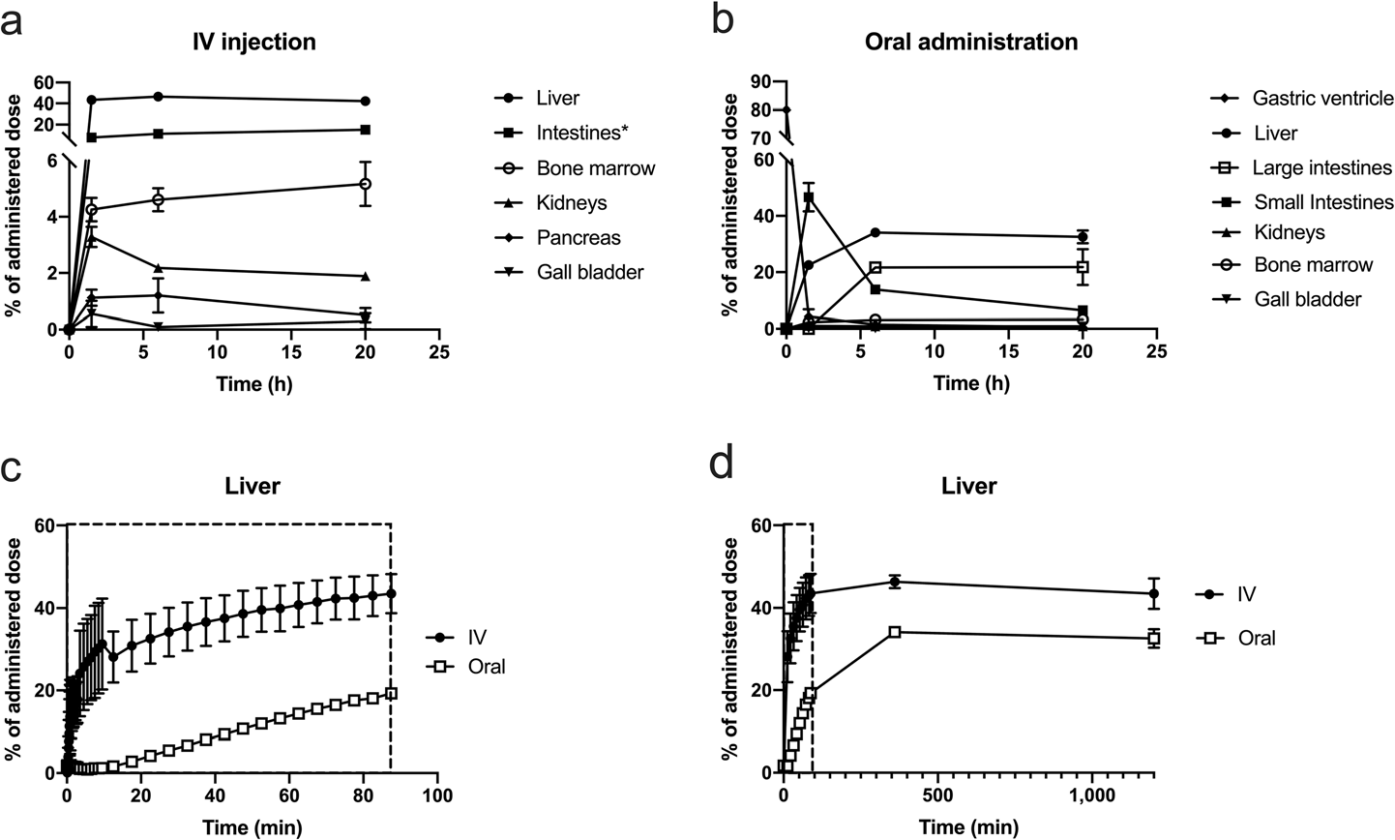
Time courses of %AD in source organs following intravenous (IV) and oral administration of ^64^Cu. Upper panels show the biodistribution of ^64^Cu after IV (**a**) and oral (**b**) administration. Lower panels show %AD in liver tissue during the initial dynamic PET/CT scan (**c**; 90 min) and including static whole-body PET scans from the entire study period (**d**; 20 h); closed circles show the time course after IV administration, open circles after oral administration. All values are given as group means ± SD. *Summation of small and large intestine

Figures 1 and 2 a-b show whole-body PET/CT and time-activity curves for the biodistribution of ^64^Cu. ^64^Cu was avidly taken up by the liver after both intravenous and oral administration with the highest concentrations reached after 6 h, where %AD in the liver was 47 ± 1.35 and 34 ± 0.01 (mean ± SD), respectively. In addition, radioactivity was observed in the red bone marrow and kidneys. Uniquely for the intravenous administration, ^64^Cu was taken up by the pancreas, intestinal walls, and salivary glands (Fig. 1). After oral administration, the biodistribution of ^64^Cu was dominated by efficient uptake by the liver and varying degrees of residual activity in the intestinal lumen. Approximately 1.5 h after administration for both groups, the variation in biodistribution between the participants was insignificant (Fig. 2a-b). Accumulation of ^64^Cu in other organs, including the brain, urinary bladder, and prostate was negligible

The hepatic uptake of ^64^Cu was rapid after intravenous administration, whereas the uptake following oral administration was delayed (approximately 12 min) by the process of intestinal absorption (Fig. 2c-d). Apart from the delay, the rate of uptake in liver tissue was comparable between the two administration forms with %AD in the liver peaking at 6 h followed by a minor decrease, most likely caused by biliary excretion and redistribution of ^64^Cu back to blood. The gallbladder was visible on the PET images in five of the six participants after 1.5 h, indicating some degree of biliary excretion at this time.

### Hepatic removal kinetics

Analysis of the hepatic removal kinetics of ^64^Cu following intravenous administration provided robust linear model-fits to the liver PET data (examples in Supplemental Figure 1). The steady-state hepatic clearance of ^64^Cu from blood into liver tissue (*K*) was 0.10 ± 0.02 mL blood/min/mL liver tissue with marginal loss of tracer into bile or blood (*k*_loss_ = 0.003 ± 0.002 min^− 1^); the hepatic volume of distribution (*V*_d_) was 36 ± 22 mL blood/mL liver tissue (mean ± SD; *n* = 3).

### Blood, urine and faeces

**Fig. 3:**
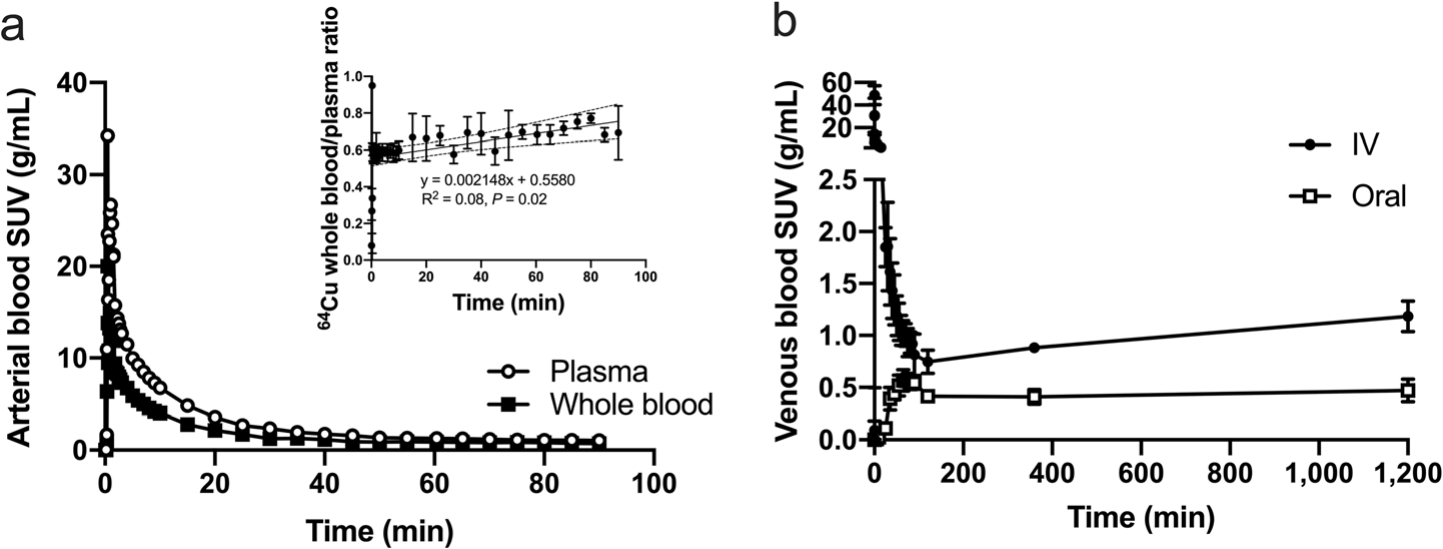
Time courses of the concentration of ^64^Cu in blood following administration intravenous (IV) and oral administration. Panel **a** shows SUV in arterial whole blood (closed circles) and plasma (open circles) following the initial 90 min after IV administration of ^64^Cu (*n* = 3; for clarity, no error bars are displayed); displayed as insert is the ratio between radioactivity concentration in whole blood and plasma. Panel **b** shows SUV in venous whole blood following IV and oral administration of ^64^Cu (*n* = 4 and *n* = 2, respectively). Values are given as group means ± SD

After intravenous administration, ^64^Cu in arterial blood was rapidly cleared from the systemic circulation; the whole-blood to plasma activity ratio was approximately 55%, increasing slowly during the initial 90 min (Fig. 3a). The venous concentration of ^64^Cu showed a similar pattern and increased slowly for the remaining study period (Fig. 3b). After oral administration, venous blood concentrations increased until approximately 1 h and then gradually subsided before a late slow increase, comparable to that observed after intravenous administration (Fig. 3b). The low radioactivity concentration in venous blood following oral administration illustrates the efficient first-pass extraction of ^64^Cu through the liver.

After intravenous administration, insignificant amounts of ^64^Cu were measured in urine (mean %AD ± SD: 0.0013 ± 0.0006) and faeces (median %AD [range]: 0.0022 [0.0002–0.0416]) during the study period (Supplemental Figure 2). Urine and faeces were not collected after oral administration. Baseline blood tests were all within normal range or near-normal (Supplemental Table 2).

### Radiation dosimetry

Participant characteristics and dosimetry data are given in Table 1; the full list of organs is given in Supplemental Table 3. After intravenous administration, the most critical organ was the liver (range 415–467 μGy/MBq), followed by the small intestines (range 168–238 μGy/MBq). After oral administration, the most critical organ was the right large intestine (range 600–925 μGy/MBq), followed by the small intestines (range 369–378 μGy/MBq), liver (range 317–335 μGy/MBq), and stomach (range 238–274 μGy/MBq). Importantly, the radiation exposure to the intestines was highly dependent on individual peristalsis and intestinal transit time as observed in participant O1, where the right large intestine received as much as 925 μGy/MBq and the left large intestine, almost nothing. In contrast, the radiation dose to the liver varied very little between the participants for both administration forms. The gender-averaged effective doses after intravenous and oral administration were 62 ± 5 and 113 ± 2 μSv/MBq (mean ± SD).

## Discussion

In this study, we report ^64^Cu PET/CT results on biodistribution, dosimetry, and hepatic removal kinetics following both intravenous and oral administration of the radiotracer in healthy humans.

### ^64^Cu biodistribution

Following absorption in the intestines, copper is transported into the portal blood circulation by the ATP7A transporter located in the basolateral membrane of the enterocytes (Nyasae et al. 2007). In the portal vein, copper is bound to albumin, in particular, and to other plasma proteins in a highly exchangeable pool (Winge 1984; Matte et al. 2017). Albumin-bound copper in systemic plasma has a half-life of 10–20 min (Janssens and Van den Hamer 1982) and is effectively extracted during the hepatic first pass (> 80%) (Cousins 1985). In the present study, uptake of ^64^Cu from the systemic circulation after intravenous administration was observed in tissues characterised by high expression of the CTR1 transporter such as the liver, pancreas, intestinal walls, and kidneys (Zhou and Gitschier 1997). After intravenous administration, ^64^Cu was not excreted in urine and only a negligible amount was detected in faeces during the 20 h observation period.

After oral administration, the biodistribution was dominated by the hepatic first pass extraction of ^64^Cu, whereas uptake in organs other than the liver, kidneys, and red bone marrow was negligible when compared with intravenous administration. Moreover, the total %AD taken up from the intestines and measured in source organs did not exceed 50%, which is in accordance with net intestinal copper absorption studies in pigs and humans (Matte et al. 2017; Turnlund et al. 1989). It should be noted that the intestinal absorption of copper is affected by the dietary composition and our results therefore only reflect conditions in the 6 h fasting state (Wapnir 1998).

In the liver, copper is incorporated in ceruloplasmin (biological half-life: 13 h) and then redistributed into the systemic circulation 2–3 days after administration, creating a second peak in blood concentration, also known as the ceruloplasmin wave (Sternlieb 1980; Marceau and Aspin 1972). In the present study, the blood concentration of ^64^Cu steadily increased after the peak following administration, reflecting copper incorporation into ceruloplasmin. The arterial blood to plasma radioactivity ratio was approximately 55% and increased over the first 90 min, possibly reflecting copper uptake in erythrocytes by the anion exchanger located in the erythrocyte membrane (Alda and Garay 1990).

### ^64^Cu dosimetry

Dosimetry estimates for intravenous administration of ^64^Cu showed that the liver was the most critical organ, followed by the small intestines. Reports of dosimetry estimates for intravenous administration of ^64^Cu differ to some extent. In the present study, radiation exposure to the liver and intestines was considerably higher and the effective dose twice of that previously reported in patients with prostate cancer (Capasso et al. 2015; Piccardo et al. 2018). This difference is likely because of different analytical approaches rather than altered biodistribution of ^64^Cu in patients with prostate cancer, compared with healthy subjects. However, our results, based on the newest phantoms and tissue weighing factors (Stabin and Siegel 2018; Basic anatomical and physiological data for use in radiological protection: reference values. A report of age- and gender-related differences in the anatomical and physiological characteristics of reference individuals. ICRP Publication 89 2002; The 2007 Recommendations of the International Commission on Radiological Protection 2007), are in agreement with the observations made by Avila-Rodriguez et al. in healthy participants (Avila-Rodriguez et al. 2017).

To our knowledge, dosimetry estimates for oral administration of ^64^Cu have not previously been reported. As expected, the radiation dosimetry of ^64^Cu after oral and intravenous administration differed significantly. While the liver was exposed to a high radiation dose after oral administration, equal or higher doses were received by the intestines due to high amounts of unabsorbed radiotracer. In this context, it is important to acknowledge that the radiation dose to the intestines depends on the individual intestinal transit time, unlike for intravenous administration; in our study, one participant received 925 μGy/MBq to the right large intestine. Consequently, the radiation dosimetry of ^64^Cu by oral administration may differ substantially between individuals, necessitating a cautious approach to total oral dose used in future studies. Based on our results, the total radiation dose received by the reference gender-averaged adult after an oral ingestion of 50 MBq ^64^Cu amounts to 5.6 mSv, ensuring less than 50 mSv absorbed by a single organ. This dose is sufficient to obtain high-quality PET images, and may still be reduced by at least 50% with new digital PET systems yielding faster time-of-flight timing resolution and higher NEMA sensitivity. Thus, ^64^Cu PET using intravenous or oral administration is suitable for studying copper metabolism in humans. In this context, it is worth noting that because of the commercial availability and long half-life of the ^64^Cu radioisotope, ^64^Cu PET can be performed also at PET centres without the necessary facilities to produce ^64^Cu.

### Copper metabolism

The use of radioactive Cu isotopes to assess copper metabolism in humans was introduced decades ago, and many studies on this topic have been published since then (Sternlieb and Scheinberg 1972; Gunther et al. 1975; Harvey et al. 2005; Czlonkowska et al. 2018). Most recently, Czlonkowska et al. showed that measurements of ^64^Cu in blood and in urine following intravenous injection accurately distinguished between patients with Wilson’s disease and heterozygote controls (Czlonkowska et al. 2018). The obvious advantage of ^64^Cu-copper PET/CT is however, the potential for assessing also the hepatic uptake, accumulation, and turnover; this includes oral administration where the biodistribution is dominated by first pass extraction by the liver, as demonstrated in the present study. In addition, the PET/CT data on the accumulation of protein-bound ^64^Cu reported in this study provides valuable knowledge to help interpret unwanted copper loss in relation to the increasing research on ^64^Cu radiopharmaceuticals (Zhou et al. 2019).

Peng et al. assessed hepatic copper kinetics in rats using ^64^Cu PET/CT (Peng et al. 2012), revealing some noteworthy differences between rodents and our human participants. For example, cardiac uptake of ^64^Cu was substantial in rodents whereas it was negligible in our human participants. Results from rodent copper studies can therefore not be easily translated to human conditions. In the present study, we were able to quantify the hepatic removal kinetics of ^64^Cu using dynamic PET/CT with measurements of arterial ^64^Cu concentration. The hepatobiliary excretion of ^64^Cu is slower than e.g. bile acids (Ørntoft et al. 2017), but importantly, the properties of the ^64^Cu isotope allow for long-term studies of copper metabolism in humans.

One of the primary therapeutic strategies in the treatment of Wilson’s disease is to inhibit absorption of dietary copper in order to reduce systemic and hepatic accumulation (Ala et al. 2007). Because of the dominant hepatic first-pass extraction of copper following ingestion, the concentration of Cu in systemic blood may not be a reliable measure when assessing how well pharmaceuticals impair absorption of copper in the intestine. While intravenous administration of ^64^Cu bypasses this first-pass metabolism, ^64^Cu PET following oral administration proves useful for assessing the enterohepatic transport of copper in vivo. Moreover, since oral ingestion of ^64^Cu reduces the invasiveness of the procedure, ^64^Cu PET/CT with oral administration would be more suitable for clinical evaluation of copper metabolism in patients with Wilson’s disease.

## Conclusion

For intravenous administration, the gender-averaged effective dose was 62 ± 5 μSv/MBq with the liver being the most critical organ. For oral administration, residual radiotracer in the gastrointestinal tract resulted in high radiation doses to the intestines, leading to an effective dose of 113 ± 1 μSv/MBq. We found that both intravenous and oral administrations of 50 MBq ^64^Cu were sufficient for sequential studies in humans, yielding images of high quality up to 20 h after administration with radiation doses of approximately 3.1 mSv and 5.7 mSv, respectively. Thus, ^64^Cu PET/CT using intravenous and oral administration represent suitable methods for assessment of copper metabolism in humans, including the intestinal absorption, hepatic removal kinetics, and subsequent redistribution of copper.

## Supplementary information

**Additional file 1.**

## Data Availability

The datasets generated and analysed during the current study are available from the corresponding author on reasonable request.

## Footnotes

1 In vivo ^64^Cu counterions/ligands exist in different oxidation states and with various exchangeable counter-ions. Throughout this paper, these ^64^Cu-labelled species are referred to collectively as “^64^Cu”.
