# Supplementary material for: Intravenous and oral copper kinetics, biodistribution and dosimetry in healthy humans studied by [64Cu]copper PET/CT
Source: EJNMMI Radiopharm Chem. 2020 Jun 18;5:15. doi: 10.1186/s41181-020-00100-1 (PMC7303253; doi:10.1186/s41181-020-00100-1)
Supplement: Supplementary file 1 — Additional file 1. [file 41181_2020_100_MOESM1_ESM.zip › 64Cu_SUPPLEMENTAL_ESM.pdf]

Supplementary Material

INTRAVENOUS AND ORAL COPPER KINETICS, BIODISTRIBUTION AND DOSIMETRY IN HEALTHY HUMANS STUDIED BY [^{64}Cu]COPPER PET/CT

Kristoffer Kjærgaard[1,2] (corresponding author; E-mail: krikje@clin.au.dk), Thomas Damgaard Sandahl[1], Kim Frisch[2], Karina Højrup Vase[2], Susanne Keiding[1,2], Hendrik Vilstrup[1], Peter Ott[1] Lars Christian Gormsen[2], Ole Lajord Munk[2].

[1]*Department of Hepatology and Gastroenterology, Aarhus University Hospital, Aarhus, Denmark;*

[2]*Department of Nuclear Medicine and PET Center, Aarhus University Hospital, Aarhus, Denmark.*

Table of contents

Figure 1. Model-fit examples 2

Figure 2. ^{64}Cu output in urine and faeces 3

Table 1. Subject characteristics for IV5-8 4

Table 2. Baseline blood tests 5

Table 3. Dose estimates for ^{64}Cu in healthy human participants 6

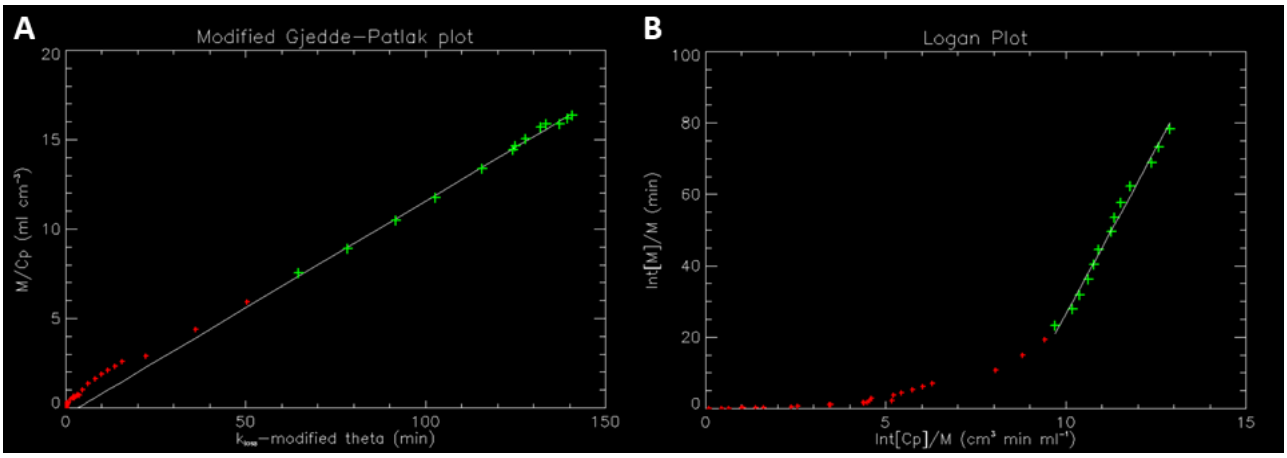

Fig. 1 Examples of model-fits from the two linearized models

A) the Gjedde-Patlak model modified to allow a small rate of loss k_{loss} from the 'irreversible' compartment, and **B)** Logan model in a healthy participant after intravenous administration of ^{64}Cu (IV2). The models are fitted to the time-activity curve of concentration in liver tissue (30-90 min), using the arterial plasma concentration as input function. Mean ± SD for the estimated model parameters are given in the main manuscript. The fitting program, iFit v.0.82, can be downloaded for free (http://liver.dk/ifit.html). Further information about installation, a short manual, and test data are now also available at the website. References for the two models are found in the main manuscript [13, 14].

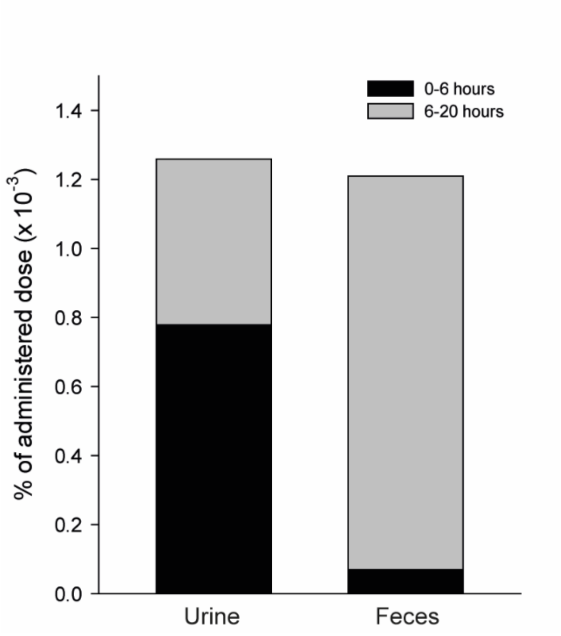

Fig. 2 Output of ^{64}Cu as % of administered dose in urine and faeces following intravenous administration

Group values are stacked and given as mean (n = 4) for urinary output and as median for faecal output (n = 4). Urine and faeces were collected from 0-6 and 6-20 h; in one participant, only faeces from 0-6 h was collected, and in another, only from 0-20 h.

ID	Gender/Age (yr)	Body Weight (kg)	Height (cm)	^{64}Cu dose (MBq)
IV5	F/23	54	163	72.9
IV6	M/21	79	184	68
IV7	M/52	95	180	74.6
IV8	F/22	88	170	73.0

Table 1 Participant characteristics for healthy participants with extra blood, urine and faecal samples obtained after intravenous administration of ^{64}Cu (IV5-IV8)

Blood tests	Median (IQR)	Normal range
ALT (U/l)	25 (5)	10-45
Total Bilirubin (µmol/l)	6.5 (4)	5-25
ALP (U/l)	88 (28)	35-105
PT	0.8 (0.4)	0.6-1.3
Albumin (g/l)	37 (7)	36-48
Urea (mmol/l)	5.3 (2)	2.6-6.4
Hemoglobin (mmol/l)	8.4 (2)	7.3-9.5
Platelets (x10^9)	233 (63)	165-400
Sodium (mmol/l)	141 (1)	137-145
Creatinine (µmol/l)	75 (15)	45-90
Zinc (µmol/l)	11 (2)	10-19
Ceruloplasmin (g/l)	0.2 (0.3)	0.15-0.45

Table 2 Baseline blood tests

Shown are median values with interquartile ranges (IQR) of blood tests assessing liver and kidney function, haematological quantities, and copper metabolism, with normal range.

Abbreviations: *ALT* = Alanine Transaminase; *ALP* = Alkaline Phosphatase; *PT* = Prothrombin Time.

Target organ	IV				Oral	
	**IV1 (M)**	**IV2 (F)**	**IV3 (M)**	**IV4 (F)**	**O1 (F)**	**O2 (M)**
Adrenals	54.5	56.9	56.8	56.1	47.3	48.9
Brain	12.2	11.4	11.4	12.1	7.9	8.3
Breasts	17.1	16.8	16.7	17.3	12.9	13.2
Esophagus	27.0	27.9	27.7	28.0	23.6	24.0
Eyes	12.2	11.4	11.4	12.1	7.9	8.3
Gallbladder	87.7	108.0	126.0	68.4	144.0	119.0
L. Large Intestine	250.0	87.1	120.0	121.0	30.4	391.0
Small Intestine	188.0	238.0	191.0	168.0	369.0	395.0
Stomach Wall	48.3	61.1	58.0	48.8	274.0	238.0
R. Large Intestine	225.0	88.7	181.0	213.0	925.0	600.0
Rectum	52.8	38.5	47.2	40.8	16.0	33.3
Heart Wall	25.7	26.2	26.0	26.4	22.8	23.1
Kidneys	137.0	128.0	133.0	132.0	66.0	72.6
Liver	415.0	467.0	462.0	446.0	317.0	335.0
Lungs	24.0	24.5	24.3	24.6	19.6	20.1
Ovaries	22.3	21.3	21.0	21.4	22.0	22.5
Pancreas	116.0	122.0	110.0	173.0	51.8	52.5
Prostate	16.5	15.5	15.5	16.0	14.1	14.6
Salivary Glands	13.1	12.3	12.3	13.1	8.6	9.0
Red Bone Marrow	36.2	34.0	35.5	32.5	27.0	24.4
Osteogenic Cells	21.5	20.3	21.0	19.7	16.1	14.9
Spleen	23.5	22.7	22.6	23.0	24.9	26.0
Testes	11.6	10.8	10.8	11.5	7.8	8.2
Thymus	17.9	17.6	17.6	18.2	13.5	13.9
Thyroid	14.8	14.2	14.2	14.8	10.2	10.7
Urinary Bladder	16.3	15.3	15.2	15.8	13.5	14.1
Uterus	23.3	22.3	21.9	22.0	22.5	24.1
Total Body	30.0	30.0	30.0	30.0	30.0	30.0
**Effective Dose**	67.6	56.2	62.0	61.3	114.0	112.0

Table 3 Gender-averaged absorbed dose estimates (µGy/MBq) and effective dose (µSv/MBq) for ^{64}Cu after intravenous (IV) and oral administration

Full list of individual organs.

Abbreviations: *F* = Female, *M* = Male.
